# Transgenic Mice Overexpressing Human *STIM2* and *ORAI1* in Neurons Exhibit Changes in Behavior and Calcium Homeostasis but Show No Signs of Neurodegeneration

**DOI:** 10.3390/ijms21030842

**Published:** 2020-01-28

**Authors:** Lukasz Majewski, Filip Maciąg, Pawel M. Boguszewski, Jacek Kuznicki

**Affiliations:** 1 International Institute of Molecular and Cell Biology in Warsaw, 4 Ksiecia Trojdena Str., 02-109 Warsaw, Poland; jacek.kuznicki@iimcb.gov.pl; 2 Postgraduate School of Molecular Medicine, Warsaw Medical University, 61 Żwirki i Wigury St., 02-091 Warsaw, Poland; 3 Laboratory of Animal Models, Neurobiology Centre, Nencki Institute of Experimental Biology of Polish Academy of Sciences, 3 Pasteur Str., 02-093 Warsaw, Poland; pmbogusz@gmail.com

**Keywords:** STIM2, ORAI1, SOCE, Alzheimer’s disease, neurodegeneration, electrophysiology, Ca^2+^ imaging, behavior, Fluoro-Jade^®^ C, amyloidosis

## Abstract

The maintenance of proper cytosolic Ca^2+^ level is crucial for neuronal survival, and dysregulation of Ca^2+^ homeostasis is found in a variety of neurological disorders, including Alzheimer’s disease. According to the “Ca^2+^ hypothesis of aging”, Ca^2+^ disturbances precede the onset of AD symptoms and lead to neurodegeneration. STIM and ORAI proteins are involved in neuronal physiological and pathological processes as essential components of the store-operated Ca^2+^ entry. Our previous data suggested that overexpression of *STIM2* and *ORAI1* might increase basal neuronal cytosolic Ca^2+^ level. We generated double transgenic mice overexpressing these two genes in neurons, expecting that the increased basal Ca^2+^ concentration will lead to premature neurodegeneration. We observed changes in Ca^2+^ homeostasis and electrophysiological properties in acute brain slices of STIM2/ORAI1 neurons. However, we did not observe any augmentation of neurodegenerative processes, as tested by Fluoro-Jade^®^ C staining and assessment of amyloidogenesis. The battery of behavioral tests did not show any signs of accelerated aging. We conclude that changes of calcium homeostasis induced by overexpression of *STIM2* and *ORAI1* had no substantial adverse effects on neurons and did not lead to early neurodegeneration.

## 1. Introduction

Store-operated Ca^2+^ entry (SOCE) is a canonical Ca^2+^ entry pathway that is activated in non-excitable cells when the ER Ca^2+^ is depleted. SOCE plays an essential role in the maintenance of cellular Ca^2+^ homeostasis and regulates a variety of processes, such as Ca^2+^ oscillations, gene expression, mitochondrial function, and apoptosis. We have shown that proteins of the SOCE pathway are expressed and functionally active also in neurons [1,2]. The Ca^2+^ sensors that convey information about the Ca^2+^ load of the ER lumen are Stromal Interaction Molecules (STIM1, STIM2). STIMs sense luminal Ca^2+^ concentration via an EF hand Ca^2+^-binding domain. In response to store depletion, they rearrange into punctate structures close to the plasma membrane, where they activate store-operated channels (SOCs), including members of the ORAI family (ORAI1-3) of Ca^2+^-influx channels [3,4]. This results in Ca^2+^ entry into the cell.

STIMs are essential players in maintaining neuronal Ca^2+^ homeostasis (reviewed in [5,6,7]). The SOCE-related function of STIMs in neurons has previously been shown by [8,9]. In EGFP-STIM1-expressing hippocampal neurons, thapsigargin caused a rapid aggregation of STIM1 in soma and dendrites [9]. Upon such STIM1 activation, a dramatic reduction in its mobility was observed by fluorescence recovery after photobleaching (FRAP) [8]. By triggering release of ER Ca^2+^ with DHPG or carbachol, agonists of metabotropic glutamate and muscarinic acetylcholine receptors, respectively, STIM1 was activated and Ca^2+^ entry occurred in dendrites. This allowed the authors to postulate that neuronal SOCE can be activated by physiological stimuli, some of which may alter the postsynaptic Ca^2+^ signaling properties [8]. Besides the regulation of SOCE, an additional role of STIM1 in inhibiting L-type voltage gated Ca^2+^ channels was demonstrated [10,11].

A second isoform of STIM proteins, STIM2, also plays a role in neuronal physiology. Notably, neurons from STIM2 knockout mice showed significantly increased survival under hypoxic conditions compared to neurons from wild-type controls [10]. The authors demonstrated that STIM2, but not STIM1, was essential for SOCE and ischemia-induced cytosolic Ca^2+^ accumulation in neurons. STIM2 knockout mice were markedly protected from neurological damage in a model of focal cerebral ischemia. Moreover, it was found that STIM2 knockout neurons had lower basal Ca^2+^ than wild-type controls. The results of Gruszczynska-Biegała et al. suggest that STIM2 regulates resting Ca^2+^ levels in the ER in rat primary cortical neurons [2,9].

Exact control of Ca^2+^ cytosolic level is necessary for cell survival and its abnormal regulation is found in a variety of disorders, including age-related diseases [11,12]. According to the most widely accepted theory, aging is a result of life-long accumulation of stochastic damages to the genomic DNA as well as to proteins and lipids, which results in the disturbance of protein expression and in an altered function of key cellular molecules (reviewed in [13]). It is justified to hypothesize that this damage is the first step that leads to the perturbation of Ca^2+^ homeostasis in aged cells. There is compelling evidence supporting an early and central role for Ca^2+^ dysregulation in the pathogenesis of Alzheimer’s disease (AD), including sporadic AD, whose etiology is not related to mutations in presenilins or APP (reviewed in [14,15,16,17]). Importantly, Ca^2+^ disturbances precede the onset of AD symptoms [18,19]. Elevated basal Ca^2+^ concentration results in excessive tau phosphorylation [20,21] and in pathological APP processing that leads to overproduction of Aβ [22]. Disturbances in Ca^2+^ signaling are found before any obvious extracellular Aβ deposition in patients with sporadic AD [23] and it has been shown that Ca^2+^ dysfunction augments Aβ formation and tau hyperphosphorylation. Elevating cytosolic Ca^2+^ level increases Aβ formation in human embryonic kidney cells [24], whereas reducing the Ca^2+^ content of the ER with thapsigargin reduces Aβ release [22]. Increased level of Ca^2+^ is observed in cells with APP knockdown or mutated presenilin [10,13]. The major change in Ca^2+^ signaling that occurs during AD appears to be an increase in the amount of Ca^2+^ released from the internal stores [25,26,27]. Even though neurons possess efficient Ca^2+^-buffering mechanisms, age-associated phenomena lead to discrete impairment of Ca^2+^ homeostasis. This, in turn, results in a decreased capacity of neurons to counteract stress, including Ca^2+^ overload. In old neurons, enhanced Ca^2+^ release from the ER or its impaired buffering function is associated with excitotoxicity and increased vulnerability to apoptosis (reviewed in [28,29]). Thus, the AD Ca^2+^ hypothesis encompasses two related phenomena (reviewed in [14,30]). The first one is that a sustained up-regulation of Ca^2+^ has pathological consequences for neurons [11,30]. The second one is that the histopathological features of AD, such as deposition of Aβ, further increase Ca^2+^ levels, thus accelerating the disease progression [14,31].

Recent results by Jadiya and colleagues demonstrate that neuronal deletion of the mitochondrial Na^+^/Ca^2+^ exchanger in 3xTg-AD mutant mice (NCLX, *Slc8b1* gene) accelerated memory decline and increased amyloidosis and tau pathology [31]. The downregulation of NCLX protein levels was also observed in postmortem brain of patients with non-familial, sporadic AD. These results provide a functional link between Ca^2+^ dyshomeostasis and AD development. It was suggested that the factors which trigger initial Ca^2+^ dysregulation, such as aging and metabolic dysfunction, in turn lead to mitochondrial dysfunction, Ca^2+^ overload and AD pathology. NCLX is known to be required for maintenance of SOCE by controlling the redox-dependent inactivation of ORAI1 [32]. On the other hand, it was shown that STIM2 was downregulated in brains of AD patients, whereas its presence was critical for the maintenance of mushroom spines in mouse models of AD [33,34].

Based on our earlier results [2] we hypothesized that mice that overexpress STIM2 and ORAI1 in neurons might exhibit early signs of neurodegeneration because of the increased basal Ca^2+^ levels. The mice with such features would confirm Khachaturian’s hypothesis of sporadic AD [19,35] and be a suitable model to study mechanisms of this disorder. In this paper we describe a new transgenic mouse line overproducing STIM2/ORAI1 in neurons. Despite the lack of obvious signs of accelerated neurodegeneration, the mice exhibit some changes in behavior and their neurons show minor impairments in basal synaptic transmission. These results highlight the importance of SOCE machinery in neuronal cells.

## 2. Results

### 2.1. Overexpression of ORAI1 and STIM2 in Neurons Leads to Altered Ca^2+^ Response in a Modified Ca^2+^ Addback Assay in CA1 Hippocampal Region

**Figure 1:**
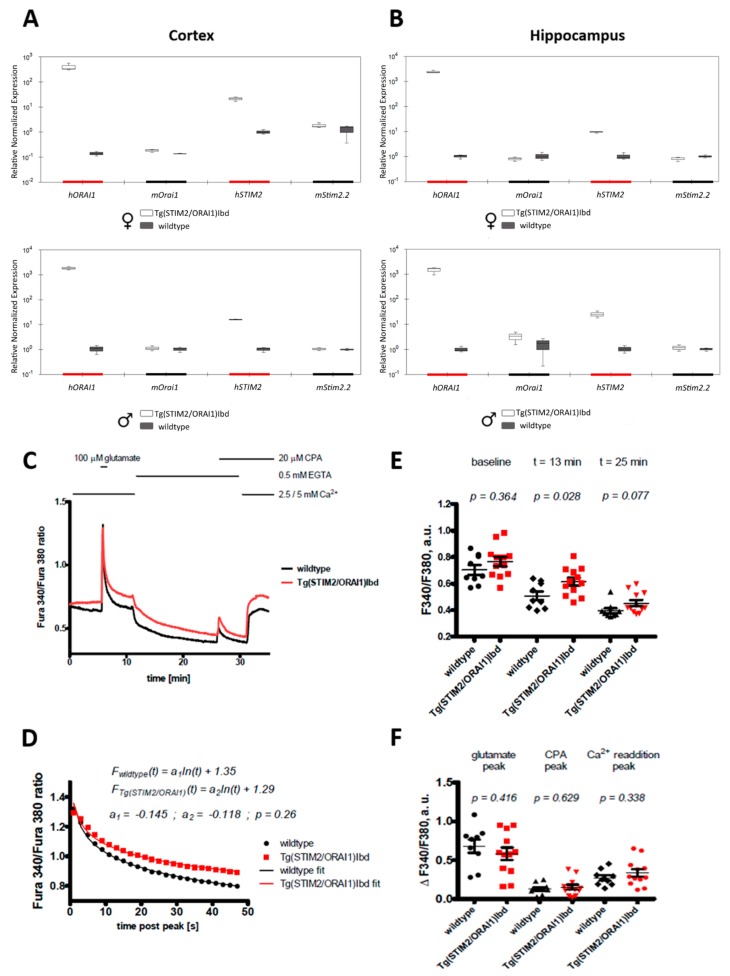
Altered Ca^2+^ response in a modified Ca^2+^ addback assay in CA1 hippocampal region from Tg(STIM2/ORAI1)Ibd line. (**A**,**B**) Expression levels of isoforms of *STIM2* and *ORAI1* in the cortex and hippocampus of male and female mice. (**C**) Averaged time-course of fluorescence signal. The measurements were performed using Fura-2 AM dye that was loaded into the CA1 neurons of the acute hippocampal brain slices. About 20 pyramidal neurons per slice (n) were analyzed; 1–2 slices from one animal were analyzed. The total number of analyzed slices was equal to 9 and 12 for wildtype and transgenic variants, respectively. (**D**) Time-course of signal decay following stimulation by glutamate that was fit by a logarithmic function. Student’s t-test was used to check statistical significance of the observed differences; *p*-values are provided above the respective charts. (**E**) Quantification of baseline F340/F380 values and following the application of Ca^2+^ chelator, EGTA; at 13th and 25th min of the measurement, a.u. (**F**) Quantification of signal amplitudes that were observed following treatment by glutamate and cyclopiazonic acid (CPA), and Ca^2+^ addition; arbitrary units (a.u.).

We generated double transgenic mice overproducing ORAI1 and STIM2 in neurons as described in Materials and Methods. Real-time quantitative PCR (qPCR) analysis was performed to check the expression levels of human transgenes in the brain of these mice and compared the level of transcripts with that of endogenous *Stim2* and *Orai1*. The results show a robust overexpression of human *STIM2* and *ORAI1* in the cortex and hippocampus of mice of both sexes (Figure 1A,B).

Acute brain slices of hippocampi that were isolated from 5-week-old wildtype and transgenic STIM2/ORAI1 mice were analyzed by fluorescent imaging. Calcium measurements and Fura-2 acetomethyl ester (AM) indicator loading were performed as described before [36]. To assess Ca^2+^ homeostasis in the STIM2/ORAI1 overproducing neurons, we used a Ca^2+^ addback assay that was modified to enable the measurement of Ca^2+^ release from the ER [37]. As shown in Figure 1C,F, stimulation by glutamate led to similar peaks in both variants tested. However, the signal decay following the pulse of glutamate tended to be slower in transgenic neurons (Figure 1D). The resulting difference in Ca^2+^ cytosolic concentration persisted during the subsequent application of a Ca^2+^ chelating agent, ethylene glycol-bis(β-aminoethyl ether)-N,N,N′,N′-tetraacetic acid (EGTA) (Figure 1C,E). Also, a moderately increased influx of calcium ions upon Ca^2+^ addback was observed in neurons overproducing ORAI1 and STIM2. Recently, we have found similar results in neurons of single transgenic line with overproduction of ORAI1 [36]. This suggests that incorporation of STIM2 did not significantly change the Ca^2+^ dynamics of neurons overproducing ORAI1 channels.

### 2.2. Impairment of Basal Synaptic Transmission in Adult Female Mice Overexpressing STIM2 and ORAI1

**Figure 2:**
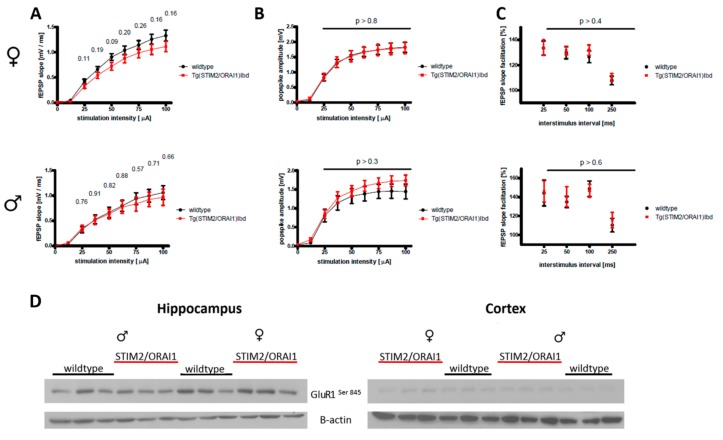
Basic electrophysiological properties of hippocampal neurons measured by local field potential recordings from acute brain slices that were isolated from 11-month-old female and male wildtype and STIM2/ORAI1 mice, upper panel: females; lower panel: males. (**A**) Input–output curves reflecting basal synaptic transmission in CA3-CA1 projection recorded from *stratum radiatum*. (**B**) Input–output curves of population spike responses recorded from *stratum pyramidale* in CA1 region. (**C**) Paired-pulse ratios of fEPSP slopes measured at different interstimulus intervals. Student’s t-test was used to check statistical significance of the observed differences; *p*-values are displayed above the respective charts. *n* = 16 wildtype and *n* = 19 STIM2/ORAI1 slices (females); *n* = 9 wildtype and STIM2/ORAI1 slices (males). (**D**) The level of GluR1 phosphorylation was analyzed by Western blot in tissue homogenates from wild type and transgenic mice of both sexes. Three mice per sex and genetic variant were analyzed.

To check basic electrophysiological properties of neurons that overproduced STIM2 and ORAI1, we recorded local field potentials in the CA3-CA1 hippocampal projection using acute brain slices. The comparison of input-output curves with those in wildtype animals revealed a modest decrease in basal synaptic transmission in slices that were isolated from transgenic females (Figure 2A, upper row). However, population spike amplitudes were virtually identical in both tested groups (Figure 2B, upper row). Similarly, we did not detect significant changes in short-term plasticity (measured by paired-pulse ratio, Figure 2C, upper row). An identical set of experiments that was performed on age-matched male mice revealed no changes in the tested parameters (Figure 2, lower row). Our results suggest that neuronal excitability is similar in STIM2/ORAI1 and wildtype hippocampi and that the minor impairments in synaptic function are specific to female mice. Following our observation of a modest impairment in synaptic transmission, we checked the phosphorylation state of GluR1, an AMPA receptor subunit, and found no major differences between the two genetic variants (Figure 2D).

### 2.3. Modest Changes in Behavior of STIM2/ORAI1 Animals

**Figure 3:**
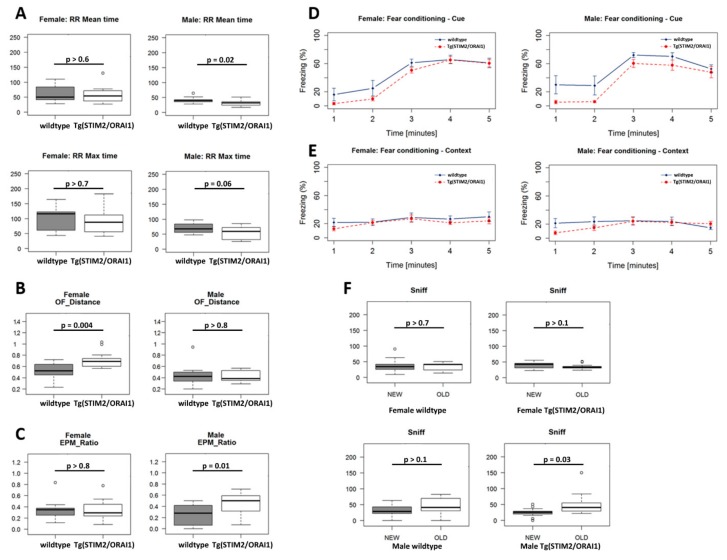
Results of the battery of behavioral tests for male and female transgenic and wildtype animals. (**A**) rotarod test (RR), mean and maximal time spent on the rotating rod; (**B**) open field test (OF), average distance moved; (**C**) elevated plus maze (EPM), ratio of entry into open vs closed arms; (**D**,**E**) fear conditioning test: cued and contextual learning, respectively; (**F**) novel object recognition (NOR) test, time spent sniffing the old and new object. Sample size: wildtype mice: n_male_ = 12; n_female_ =12; Tg(STIM2/ORAI1)Ibd mice: n_male_ = 13; n_female_ = 15.

To check whether the observed alterations in Ca^2+^ homeostasis and the moderate impairment in synaptic transmission translates into changes in behavior of adult mice (6-month-old), we performed a battery of tests. Female transgenic mice exhibited enhanced willingness to explore the arena in Open Field test, which could indicate increased exploratory drive or decreased anxiety (Figure 3B). Transgenic males spent less time on the rotating cylinder in the RotaRod test (Figure 3A,B). Also, male transgenic animals tended to spend more time in the open arms during the elevated-plus maze (EPM) test (Figure 3C), which suggests lower anxiety. Interestingly, whereas cued and contextual learning abilities were comparable across sexes and genetic variants (Figure 3D,E), male transgenic mice exhibited less freezing during the first 2 min of the cued conditioning protocol, before the stimulus was applied. This observation may correspond to the observed lower anxiety levels in the EPM test. In the Novel Object Recognition test, we observed differences in the discrimination of the old and novel objects in the case of male mice, but not in females (Figure 3F). Altogether, our results suggest that overproduction of STIM2 and ORAI1 had a modest effect on the behavior of animals. Apparently, the observed changes in Ca^2+^ homeostasis and electrophysiological properties did not have a significant impact on learning and memory of transgenic mice and did not trigger premature pathology.

### 2.4. No Features of Neurodegeneration in Tg(STIM2/ORAI1)Ibd Mice

**Figure 4:**
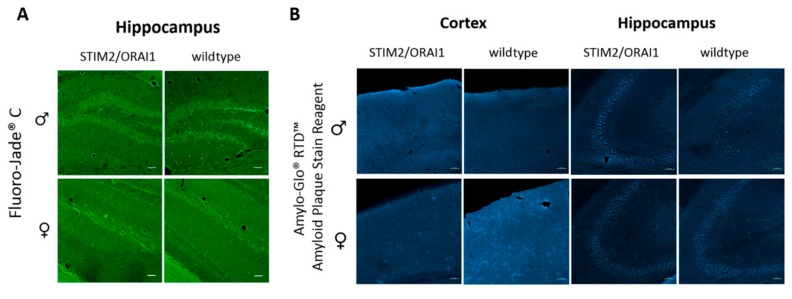
Results of immunostaining against markers of neurodegeneration. (**A**) Fluoro-Jade^®^ C and (**B**) Amylo-Glo RTD immunostaining of brain slices from wild type and transgenic mice of both sexes. We observed no signs of neurodegeneration; three mice per sex and genetic variant were analyzed, shown is only one set of data. The scale bars show 50 μm.

Following the initial idea of the project, we wondered whether overexpression of *STIM2* and *ORAI1* genes would trigger accelerated neurodegenerative processes because of the expected changes in Ca^2+^ homeostasis. To evaluate this hypothesis, we performed staining with the Fluoro-Jade^®^ C dye that specifically labels degenerating neurons (Figure 4A). Additionally, potential deposition of amyloid plaques was checked by immunostaining (Figure 4B). No differences between wildtypes and STIM2/ORAI1 mice were observed, which suggests that overproduction of these two proteins had no effect on neurodegenerative processes.

## 3. Discussion

Accumulating evidence suggests that STIMs and ORAIs, the key proteins responsible for SOCE, play an important role in neuronal physiology. To explore further the role of neuronal SOCE (nSOCE), we have recently created several transgenic mouse lines that overproduce SOCE proteins specifically in brain neurons. Using these mice, we have shown that the activities of ORAI1 and STIM1 may regulate basal synaptic transmission and synaptic plasticity [38,39]. Together with studies by other groups [40,41,42,43,44], our results show that SOCE proteins play a role in fundamental neuronal processes. The proper functioning of synaptic transmission and plasticity is in part conveyed through intricate control of Ca^2+^ dynamics in neurons [45]. We have previously found that in cultured cortical neurons, the overproduction of STIM2 together with ORAI1 resulted in an increased basal cytoplasmic Ca^2+^ concentration [2]. Along the lines of so-called ‘Ca^2+^ hypothesis of aging’, this observation prompted us to create a transgenic mouse line with neuronal-specific overproduction of these two proteins. This could serve as a useful model to validate whether the expected elevated Ca^2+^ level in neurons facilitated premature neurodegeneration, a hallmark of AD and other neurological disorders [19].

In contrast to our findings in cultured neurons [2], Ca^2+^ imaging that was performed on hippocampal slices isolated from double transgenic STIM2/ORAI1 mice did not reveal significant changes in basal cytosolic Ca^2+^ level (Figure 1C,E). This may result from the differences between biological models that were used in the original and the current study (neuronal cultures [2] vs ex vivo tissue—this study) or from the existence of compensatory mechanisms that are often observed in transgenic animals [46]. Nevertheless, our Ca^2+^ imaging experiments showed an altered Ca^2+^ response in neurons following a strong physiological stimulus (the application of glutamate). The changes were akin to the ones that we have recently observed in single transgenic ORAI1 mice [36]—a slower relaxation of the signal following glutamate peak and the persistence of increased cytosolic Ca^2+^ level in Ca^2+^ chelating conditions. Based on the mechanism proposed by Chen Engerer at al [47], this supports our hypothesis that overproduction of ORAI1 may have altered the refilling of ER Ca^2+^ stores. Interestingly, the co-expression of STIM2 together with ORAI1 seemed to have little effect on the observed changes in Ca^2+^ homeostasis. Indeed, the previously described Ca^2+^ dynamics in hippocampal neurons overexpressing STIM2 alone was similar to that of wild-type neurons, with the exception of elevated Ca^2+^ influx in the ‘Ca^2+^ re-addition protocol’ that is used to assess the magnitude of SOCE [36]. Field potential recordings have revealed minor impairments in basal synaptic transmission of double transgenic female STIM2/ORAI1 mice (Figure 2A). This change was absent in male STIM2/ORAI1 mice.

In our transgenic model, STIM2/ORAI1 mice, the alterations in Ca^2+^ homeostasis that we have observed did not correlate with detectable signs of neurodegeneration. Fluoro-Jade^®^ C staining (which specifically detects degenerating neurons) and immunostaining for Aβ revealed no detrimental changes in hippocampal neurons from these mice (Figure 4). Despite modest impairments in synaptic transmission, we did not observe any changes in the phosphorylation state of the Ser845 GluR1 subunit of the AMPA receptor (Figure 2D). Likewise, transgenic animals performed similarly in most behavioral tests that assess cognitive skills. However, male animals of the transgenic lines were more willing to enter open arms during elevated plus maze test, which could be a sign of decreased anxiety. Also, transgenic males spent less time on the rotating cylinder in the RotaRod test. This may indicate problems with motor coordination. Moreover, male mice of STIM2/ORAI1 manifested altered recognition of the novel object. Together with our previous report [38], these findings provide further indications that some actions of SOCE proteins may be sex-dependent.

Despite the lack of expected premature neurodegeneration in the STIM2/ORAI1 mice, our novel model can be a useful tool for further studies of the role of SOCE proteins in neurons. This is especially relevant in the context of conflicting evidence of STIM2 actions in neuronal pathophysiology [10,31,33,48,49,50,51,52], the involvement of ORAI1 in neurotransmission [53] and the growing body of evidence that links Ca^2+^ homeostasis to AD-related neurodegeneration.

## 4. Materials and Methods

### 4.1. Chemicals and Antibodies

Cyclopiazonic acid (CPA) was from Sigma (St. Louis, MO, USA) (catalog no. C1530). Fura-2 AM was from Thermo Fisher Scientific (Waltham, MA, USA) (catalog no. F1221). Fluoro-Jade^®^ C was from Millipore (catalog no. AG325, lot 3170812). Amylo-Glo^®^ RTD™ Amyloid Plaque Stain Reagent was from Biosensis (Thebarton, Australia) (catalog no. T3-300-AG, lot BA01-30-300 AGTK120419). Mounting medium Entellan^®^ new was from Millipore (Burlington, MA, USA) (catalog no. 107961, lot HX42534561). Polyclonal rabbit monoclonal antibody against phospo-GluR1 Ser845 was from Millipore (catalog no. EPR2148; lot 2377032). Monoclonal antibody calcineurin (CaN) was from Sigma (St. Louis, MO, USA) (catalog no. C1956; lot 094M4820V). The housekeeping protein monoclonal mouse β-actin antibody was from Sigma (St. Louis, MO, USA) (catalog no. A54418, lot 122M4782). Secondary anti-rabbit and anti-mouse antibodies were conjugated with horseradish peroxidase (HRP; Sigma catalog no. A9169 lot 117M4808V and A9044 lot 055M4818V).

### 4.2. Animal Care

The mice were housed under standard conditions on a 12 h/12 h light/dark cycle with food and water available ad libitum. All of the animal experiments were approved by the Local Commission for the Ethics of Animal Experimentation no. 1 in Warsaw (approval no. 416/2017, 21 November 2017) and performed in accordance with the European Communities Council Directive (63/2010/EEC, 22 September 2010).

### 4.3. Generation of FVB/NJ–Tg(STIM2/ORAI1)Ibd Transgenic Mice

A coding sequence of human STIM2 from pEX-CMV-SP-STIM2(15-746) plasmid (a gift from Tobias Meyer—Addgene plasmid # 18868) [54] was cloned to the Thy-1.2 expression cassette [55,56]. The STIM2 coding region and flanking Thy1.2 sequence were sequenced to confirm that no point mutations were introduced during the cloning steps. Next, the Thy1.2-STIM2 construct was digested with *PvuI* and *NotI* to excise plasmid sequences, and the linearized fragment was gel-purified and used for standard pronuclear microinjection [57] and injected into pronuclei of fertilized eggs that were derived from FVB/NJ. The procedure was performed in the Laboratory of Animal Models at the Nencki Institute of Experimental Biology (Warsaw, Poland). Quantitative Real-Time PCR confirmed specific expression of the transgene in the brain within the established FVB/NJ-*Tg*(STIM2)Ibd lines. Generation of the FVB/NJ-*Tg*(ORAI1)Ibd line was described elsewhere [38]. Double transgenic mice were generated by crossing homozygotes of male FVB/NJ-*Tg*(STIM2)Ibd with the female FVB/NJ-*Tg*(ORAI1)Ibd. To identify transgenes, genotyping was performed with the following primers: Thy_Forw_genotype 5′-TCTGAGTGGCAAAGGACCTTAGG-3′, ORAI1_Rev_genotype 5′-TGGTCCTGTAAGCGGGCAAAC-3′, STIM2_Rev_genotype 5′-GCTGCTTATTCTGGCAACACTTGG-3′.

### 4.4. Real-Time PCR Analysis for Neuronal Confirmation of Expression of Both Transgenes

Adult mouse (postnatal day 180 [PD180]) brain structures (i.e., hippocampus, cortex) were digested using the RNeasy Lipid Tissue Mini Kit (Qiagen, Hilden, Germany) followed by DNase (Qiagen) treatment according to the manufacturer’s protocols. RNA templates (500 ng) were used to synthesize first-strand cDNA using a reverse transcription kit (iScript, Bio-Rad: 170–8841, USA). The real-time PCR reactions were performed in duplicate for each sample using SsoAdvanced Universal SYBR Green Supermix (Bio-Rad:1725274; Hercules, CA, USA) with the following specific gene primers for human STIM2: 5′-TTGCTGGAGGAGTTGATGAC-3′; 5′-CTGCTGCTTCTGGCTAATG-3′ (Sigma), human ORAI1 5′-CTCTCCGGCTTCGCCAT-3′; 5′-ACAGCCACCAGCACTGT-3′ (Sigma), mouse Orai1 5′-ATGAGCCTCAACGAGCACT-3′; 5′-GTGGGTAGTCATGGTCTG-3′ (Sigma), and mouse STIM2.2 5′-GGACGAGGCAGAAAAAATTAAAAAG-3′; 5′-CACGTGGTCAGCTCAGAGAG-3′ (Sigma). Uba-2 was used for normalization with the following primers: 5′-GGCTTGATAGTGTTGGAAGGA-3′; 5′-CTTGGGTTTGGCTGCTTATTC-3′ (Sigma). The specificity of the reactions was determined based on dissociation curve analysis. Relative gene expression was calculated using the 2^-ΔΔCt^ method using Bio-Rad CFX Maestro 1.1 Software (Bio-Rad, Hercules, CA, USA), USA). The reactions were performed using the CFX Connect™ Real-Time PCR Detection System (Bio-Rad). Three individuals per variant per sex were analyzed.

### 4.5. Protein Isolation and Immunoblotting

Fresh brain tissues (PD 180) were homogenized in a buffer that contained 50 mM Tris (pH 7.5), 150 mM NaCl, 1% TX-100, 0.5% sodium deoxycholate, and 1 mM ethylenediaminetetraacetic acid (EDTA) with protease inhibitor mixture (Roche Applied Science, Penzberg, Germany) and phosphatase inhibitor mixtures 2 and 3 (Sigma) using a glass–glass homogenizer. Protein extracts were separated on 10% SDS-polyacrylamide gels, and proteins were detected by Western blot with anti-phospho-GluR1 Ser845 antibody (1:1000; Millipore, catalog no. EPR2148; lot 2377032), mouse anti-calcineurin antibody (1:10000; Sigma, catalog no.C1956; lot 094M4820V), and anti-β-actin antibody (1:10000, Sigma; catalog no. A54418, lot 122M4782). Secondary anti-rabbit and anti-mouse antibodies were conjugated with HRP (1:10000; 1:5000 respectively, Sigma; catalog no. A9169 lot 117M4808V and A9044 lot 055M4818V). Three individuals per variant per sex were analyzed.

### 4.6. Perfusion of Mice and Brain Sectioning

Adult mice (postnatal day 180 (PD180)) were deeply anesthetized with an intraperitoneal injection of ketamine (Biowet, Poland) with xylazine (Vetoquinol Biowet, Poland) and transcardially perfused with a 0.1 M phosphate-buffered (PB) solution (pH 7.4), followed by 4% paraformaldehyde (PFA) in 0.1 M PB (pH 7.4). Dissected brains were postfixed overnight in 4% PFA and further cryoprotected overnight with 30% sucrose. The brains were coronally sectioned at 20 μm with vibratome Compresstome (Precisionary Instruments, Greenville, SC, USA).

### 4.7. Immunohistochemistry

Fluoro-Jade^®^ C (staining of degenerating neurons) and Amylo-Glo^®^ RTD™ (staining of amyloid plaques in tissue sections) procedures were performed according to the manufacturer’s protocols. Briefly, the brain sections were mounted on Super Frost Ultra Plus slides (Thermo Scientific, Hungary), dried at 50–60 °C on a slide warmer for at least 30 min to 1hr for good adhesion. Next, in case of Fluoro-Jade^®^ C staining, slides were immersed in a basic alcohol solution comprising 1% sodium hydroxide in 80% ethanol for 5 min. They were then transferred to a new Coplin jar containing freshly prepared 70% ethanol for 2 min and next rinsed for 2 min with 0.1M PB (phosphate buffer). Following incubation in 0.06% potassium permanganate solution for 10 min for fluorescent background blocking and contrast optimization, the slides were transferred for 10 min to a 0.0001% solution of the Fluoro-Jade^®^ C dissolved in 0.1% acetic acid. The slides were then rinsed three times in 0.1 M PB for 1 min, air dried on slide warmer at 50 °C for at least 5 min. Next, dry slides were cleared by brief immersion in xylen (POCH, Poland) and coverslipped with non-aqueous mounting medium Entellan^®^ new (Millipore, Germany). In case of Amylo-Glo^®^ RTD™ procedure, the dried slides were transferred into a 70% solution of ethanol for 5 min, then rinsed in 0.1 M PB for 2 min and incubated for 10 min in the prepared 1× Amylo-Glo^®^ RTD™ staining solution. Next the slides were transferred into 0.9% saline solution for 5 min, briefly rinsed in 0.1 M PB, air dried, dehydrated through a graded series of ethanol concentrations, cleared in xylene (POCH, Poland) and coverslipped with Entellan^®^ new (Millipore, Germany). Images were acquired with a 10× objective on Zeiss Airyscan detector. Three individuals per variant per sex were analyzed.

### 4.8. Behavioral Analysis

Behavioral experiments were performed with a group of 25 males (12 wild-types, 13 transgenes) and 27 females (12 wild-types, 15 transgenes, age PD180) according to the following protocol: Rotarod test (RR), open field test (OF), elevated plus maze (EPM), fear conditioning, and novel object recognition test (NOR). 

The tests were described extensively elsewhere [38,39]. In brief, in the rotarod test the mice were forced to maintain their balance on a rotating rod (30 mm diameter) that was placed 20 cm above the apparatus floor. The time (latency) it took for the mouse to fall from the rod (0–40 rotations per minute (rpm)) in a 300 s session was recorded. In the open-field test, the animals were placed in the middle of a circular arena (64 cm diameter, 30 cm high walls) and their activity was recorded for 10 consecutive minutes. In EPM, testing was performed using an apparatus that consisted of two opposing open arms and two opposing closed arms, (both 35 cm × 5 cm). The open and closed arms were arranged perpendicular to each other, with a central platform at the intersection of the arms. Animal movements were recorded for 5 consecutive minutes.

In fear conditioning test, a speaker was used to deliver the acoustic conditioned stimulus (CS). Sensory stimuli were adjusted to generate two contexts (context A and context B). For context A, a house light that was mounted on the ceiling of the cage was illuminated, and the room lights remained on. For context B, the room and chamber house lights were turned off. The ventilation fans were turned off and a 60 W red light provided illumination. The animals underwent three phases of training: fear conditioning, cue testing, and context testing. For fear conditioning, the animals were placed in the conditioning chambers in context A. They were exposed to five tones followed by foot shocks, with a 60 s interval. Twenty-four hours after the conditioning session, the mice underwent cue testing in a novel context (context B). Two minutes after being placed in context B, a tone was delivered. Twenty-four hours after cue testing, the animals were subjected to context testing. They were placed in the same context as in the fear conditioning phase (context A) for 300 s. Fear-related behavior (i.e., freezing) in response to the tone and context stimuli during training and testing was assessed by an automated video-based analysis system [58].

The novel-object recognition test was conducted in boxes (40 cm × 40 cm × 30 cm) with even lighting conditions (70 lux). Two laboratory glass bottles (6 cm diameter, 8 cm height) were used as Objects A, and a door stopper (6 cm diameter, 7 cm height, round in shape, stainless steel) was used as Object B. On day 1 of the tests, a habituation trial was performed. The mice were allowed to explore the empty test box for 5 min. The learning trial was conducted twenty-four hours after the habituation trial. The animals were placed in the test box with two identical Objects A that were positioned in opposite corners. The mouse was allowed to explore both for 5 min before it was placed back into its home cage. After 24 h, the mouse was placed in the test box with familiar Object A and novel Object B. The mouse was allowed to explore the objects for 5 min. The videos were recorded and analyzed using a video-tracking system (EthoVision, Noldus) to extract the behavioral data.

The differences between the groups were evaluated using the nonparametric Mann–Whitney U test. The data are expressed mean ± SEM. Data preparation and analysis were performed using R software (https://www.r-project.org).

### 4.9. Brain Slice Preparation, Ca^2+^ Imaging, and Electrophysiology

12 ± 1 month-old and P25 ± 5 mice were used for electrophysiological experiments and Ca^2+^ imaging, respectively. Following cervical dislocation, hippocampi from the right hemisphere were retrieved and 350 μm thick sections were prepared according to the protective recovery method and as described previously [36]. For Ca^2+^ imaging experiments, CA1 pyramidal layer was labeled with the use of Fura-2 AM indicator and a modified Ca^2+^-addback assay was performed as described before [36]. The concentration of glutamate, EGTA and CPA were 100 μM, 2.5 mM and 20 μM, respectively. The authors wish to note that the imaging experiments that are described in the present paper and in the paper [36] were performed simultaneously, but the results concerning the double transgenic STIM2/ORAI1 line were not included in the latter work. The wildtype controls are the same for both sets of data, therefore, they overlap with our previously published work.

Local field potential recordings were performed in a submerged chamber that was filled with artificial cerebrospinal fluid (aCSF) that contained, in mM: 126 NaCl, 2.6 KCl, 20 glucose, 1.25 NaH_2_PO_4_, 25 NaHCO_3_, 1.5 MgSO_4_, and 2.5 CaCl_2_. The pH was adjusted to 7.3–4 at room temperature with O_2_/CO_2_ 95%/5% (*v/v*) mixture (carbogen) and HCl, and the osmolality was 315 ± 5 mOsm/kg H_2_0. aCSF was carbogenated throughout the recordings and heated with an in-line heater (catalog no. 64-0102, controlled by a TC-324B temperature control unit, Warner Instruments) to maintain the temperature in the recording chamber at 25 ± 1 °C. The rate of solution flow was 7 mL/min. To obtain synaptic responses, Schaffer collaterals were stimulated with a bipolar electrode (catalog no. CBARC75, FHC, USA). Stimuli of increasing intensity (0–100 μA, every 12.5 μA) were delivered with an ISO-200 stimulator (Circlelabs) at a frequency of 0.1 Hz. Field excitatory postsynaptic potentials (fEPSPs) were recorded in the stratum radiatum and stratum pyramidale of the CA1 area of the hippocampus using standard Ag/AgCl electrodes that were connected to CV-7B headstages (Molecular Devices). Glass micropipettes that were mounted on the headstages were pulled from borosilicate glass (Warner Instruments, 0.86 mm inner diameter, 1.50 mm outer diameter) with the use of a horizontal puller (P-1000, Sutter Instruments). Resistance of the pipette tips was between 2 and 4 MΩ when filled with aCSF.

The signals were amplified with Multiclamp 700B amplifier and acquired at 20 kHz with a Digidata 1550B acquisition card (Molecular Devices) using a 3 kHz low-pass filter. Prior to analysis, the signals were filtered at 1 kHz. To calculate popspike activity, a built-in function of the AxoGraph program (developed by John Clements) was used. To analyze synaptic activity, initial linear part of the slope of fEPSP was calculated in Clampfit (Molecular Devices). Further data analyses were performed in Microsoft Excel and GraphPad Prism 5 (USA).

## 5. Conclusions

Together with our previously published study, our data show that overproduction of SOCE proteins influence neuronal activity and may exert sex-dependent effects. Despite the observed impairments in synaptic transmission in transgenic female STIM2/ORAI1 mice and minor behavioral alterations in transgenic male animals, we did not detect any signs of accelerated neurodegeneration.

## Abbreviations

Aβ	amyloid beta
aCSF	artificial cerebrospinal fluid
AD	Alzheimer’s Disease
AM	acetomethyl
AMPA	α-amino-3-hydroxy-5-methyl-4-isoxazolepropionic acid
AMPK	adenosine monophosphate-activated protein kinase
APP	amyloid precursor protein
a.u.	arbitrary units
CPA	cyclopiazonic acid
DHPG	(*S*)-3,5-Dihydroxyphenylglycine
EDTA	ethylenediaminetetraacetic acid
EGTA	ethylene glycol-bis(β-aminoethyl ether)-N,N,N′,N′-tetraacetic acid
EPM	elevated plus maze
ER	endoplasmic reticulum
fEPSP	field excitatory postsynaptic potentials
FRAP	fluorescence recovery after photobleaching
GluR1	glutamate receptor 1
NCLX	mitochondrial Na^+^/Ca^2+^ exchanger
NFAT	nuclear factor of activated T-cells
NOR	novel object recognition
nSOCE	neuronal store-operated calcium entry
OF	open field
PCR	polymerase chain reaction
PB	phosphate buffer
qPCR	quantitative polymerase chain reaction
RR	rotarod
sAD	sporadic Alzheimer’s disease
SDS	sodium dodecyl sulfate
SOCE	store operated calcium entry
SOCs	store-operated channels
STIM	stromal interaction molecule
Tg	transgenic
TX-100	Polyethylene glycol *p*-(1,1,3,3-tetramethylbutyl)-phenyl ether
